# The influence of lumbo-sacral transitional vertebrae in developmental dysplasia of the hip: a matched pair analysis

**DOI:** 10.1038/s41598-023-37208-8

**Published:** 2023-06-20

**Authors:** Luis Becker, Christian Hipfl, Friederike Schömig, Carsten Perka, Sebastian Hardt, Matthias Pumberger, Vincent Justus Leopold

**Affiliations:** Center for Musculoskeletal Surgery, Charité – University Medicine Berlin, Charitéplatz 1, 10117 Berlin, Germany

**Keywords:** Musculoskeletal system, Bone, Skeleton

## Abstract

Lumbo-sacral transitional vertebrae (LSTV) are the most common congenital alteration of the lumbo-sacral junction and known to significantly influence pelvic anatomy. However, the influence of LSTV on dysplasia of the hip (DDH) and the surgical treatment by periacetabular osteotomy (PAO) remains unknown. We retrospectively examined standardized standing anterior–posterior pelvic radiographs of 170 patients in 185 PAO procedures. Radiographs were examined for LSTV, lateral-central-edge-angle (LCEA), Tönnis-angle (TA), femoral-head-extrusion index (FHEI), and anterior-wall-index (AWI) and posterior-wall-index (PWI). Patients with LSTV were compared to an age- and sex-matched control group. Patient-reported outcome measurements (PROMs) were evaluated pre- and in the mean 63.0 months (range 47–81 months) postoperatively. 43 patients (25.3%) had LSTV. Patients with LSTV had significantly greater PWI (*p* = 0.025) compared to the matched control group. No significant differences were seen in AWI (*p* = 0.374), LCEA (*p* = 0.664), TA (*p* = 0.667), and FHEI (*p* = 0.886). Between the two groups, no significant differences were detected in pre- or postoperative PROMs. Due to the increased dorsal femoral head coverage in patients with LSTV and DDH compared to patients with sole DDH, a more pronounced ventral tilting might be performed in those patients with prominent posterior wall sign to avoid anterior undercoverage, which is a significant predictor for premature conversion to hip arthroplasty after PAO. However, anterior overcoverage or acetabular retroversion must be avoided due to the risk of femoroacetabular impingement. Patients with LSTV reported similar functional outcomes and activity after PAO as the control group. Therefore, even for patients with concomitant LSTV, which are frequent with one-fourth in our cohort, PAO is an efficient treatment option to improve clinical symptoms caused by DDH.

## Introduction

Changes in the bony anatomy of the pelvis, including the lumbosacral junction, significantly influence pelvic geometry and acetabular version. These alterations play a critical role in the development of hip and low back pain^1–3^. Developmental dysplasia of the hip (DDH) is one of the most frequent pelvic alterations, with a reported prevalence ranging from 0 to 40%. It is characterized by reduced coverage of the femoral head by the acetabulum, often leading to anterolateral femoral head coverage insufficiency^4,5^. The deficient coverage of the femoral head could result in pathological contact pressures in the area of the acetabular rim, which leads to overload of the cartilage and labrum in this area and, ultimately, to the development of secondary osteoarthritis of the hip^6,7^. Periacetabular osteotomy (PAO) is the gold standard for joint-preserving surgical therapy in DDH and shows excellent results both radiologically and clinically^8–10^.

In the spino-pelvic junction lumbo-sacral transitional vertebrae (LSTV) are one of the most frequent alterations with a reported prevalence of 4–36.5%^11^. LSTV result from an enlargement of the transversal process and lumbarization of the most cranial sacral vertebra or sacralization of the most caudal lumbar vertebra^11,12^. For LSTV, an influence on pelvic incidence, which defines pelvic geometry is described and therefore an interaction with acetabular anatomy as well as DDH is plausible^2^.

While a high co-prevalence of LSTV in DDH patients has been reported, there still is a lack of literature of LSTV on femoral head coverage in patients with DDH scheduled for PAO^13^. Thus, our study’s aim was to investigate the influence of LSTV on femoral head coverage and on the surgical therapy of DDH with PAO.

Therefore, we hypothesized:

1. In patients with LSTV, changes of pelvic geometry have an influence on femoral head coverage in DDH patients.
2. Changes in pelvic geometry in patients with LSTV lead to significant differences in femoral head coverage after PAO.
3. Patients with LSTV and DDH differ from patients with sole DDH in clinical patient-reported outcome measures (PROMs) after PAO.

## Methods

### Study cohort

We performed a retrospective study of patients with primary diagnosis of symptomatic DDH after approval by the local ethics board of Charité University Hospital Berlin (EA2/232/19). Informed consent was waived of the aforementioned ethics board due to retrospective study design. Study was performed according to the declaration of Helsinki and STROBE-criteria. One hundred eighty-three consecutive patients who underwent PAO at our institution between January 2015 and June 2017 were included. Exclusion criteria for this study included cases with insufficient or missing pre- or postoperative radiological imaging, primary diagnoses other than DDH (e.g., acetabular retroversion), or previous surgery on the ipsilateral hip joint. Six hips had to be excluded due to primary diagnosis of acetabular retroversion and eleven due to insufficient pre- or postoperative imaging, yielding a total of 170 patients with 185 PAOs to be included.

### PAO procedure

The surgical procedure was performed adapted to the description by Ganz et al. by four attending specialized hip surgeons^14^. The operation was performed using an anterior approach as described previously^15^. Restoration of the three-dimensional coverage with a lateral Wiberg mean angle (LCEA) of 30° was aimed for with persistency of > 10° of acetabular anteversion. This was achieved by tilting the osteotomy fragment ventrally and laterally under intraoperative posterior-anterior fluoroscopic control. Thereafter, fixation of the acetabular fragment using cortical screws or Kirschner-wires and layered wound closure were performed. The postoperative regimen included tip-touch partial weight-bearing for a period of 6 weeks postoperatively. After the 6th week, weight-bearing was increased to half the patient's body weight from the seventh to the 10th postoperative week. After the 10th week, weight bearing was gradually increased to full weight bearing by 3 months postoperatively. The range of motion of the operated hip joint was not restricted.

### Image analysis

**Figure 1:**
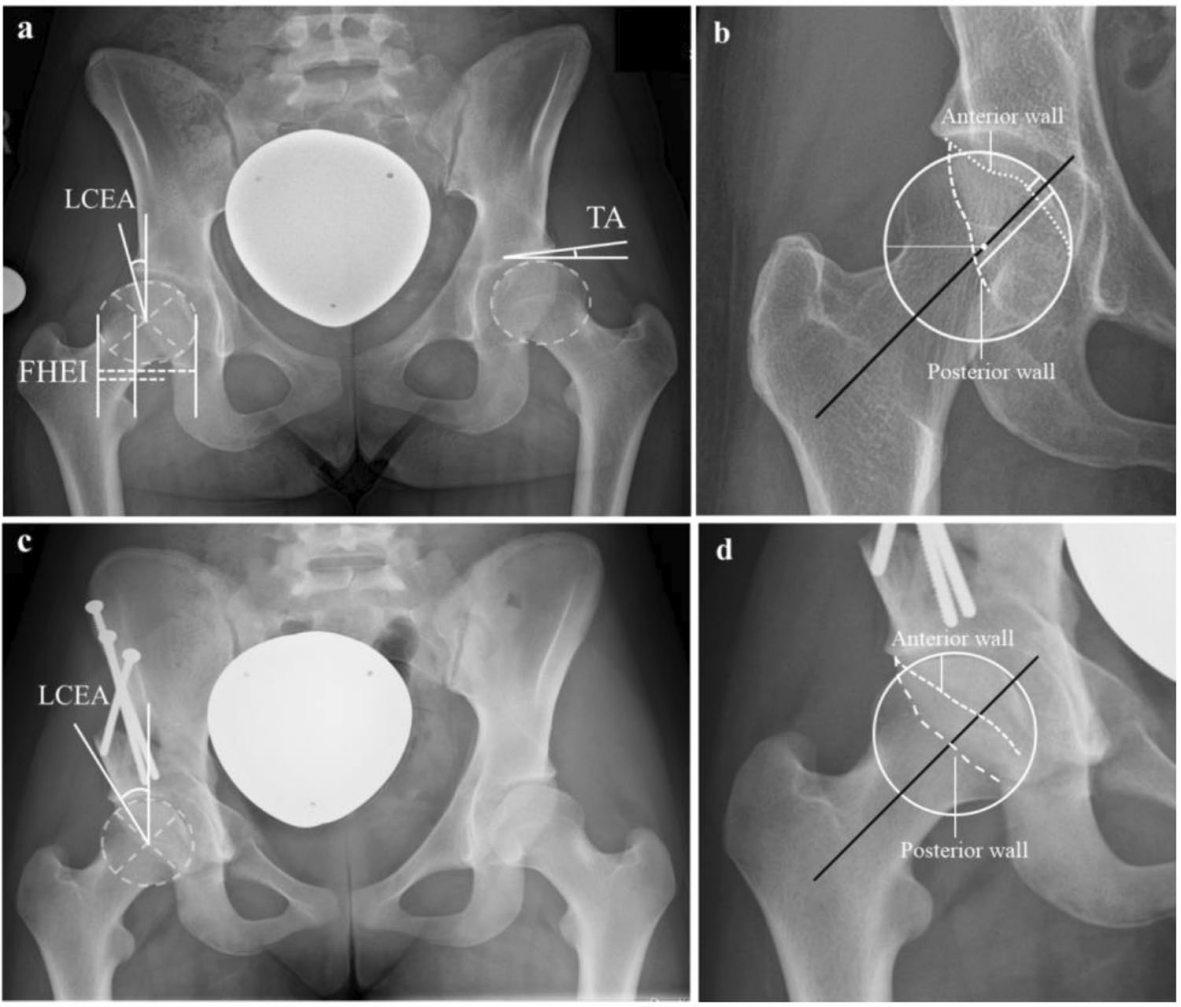
Measurement of the radiological parameters on a.p. pelvic radiographs in standing position pre- and postoperatively. (**a**) illustrates the measurement of the parameters Tönnis angle (TA), lateral-central-edge angle (LCEA) and femoral-head-extrusion-index (FHEI) on the a.p. pelvic radiographs of a patient with LSTV Castellvi IIIb preoperatively. (**b**) shows the measurement of the anterior and posterior wall index preoperatively. (**c**) depicts the measurement of LCEA postoperatively. (**d**) shows the measurement of anterior and posterior wall index postoperatively.

All patients received standardized standing anterior–posterior standing pelvic radiographs. DDH-related abnormalities were defined as an LCEA less than 25°, a Tönnis angle (TA) greater than 10°, or a femoral head extrusion index (FHEI) exceeding 25%^16^. Furthermore, AWI and PWI were determined as measures of anterior and posterior coverage^17^. Radiological parameters relevant for DDH were measured both pre- and postoperatively before hospital discharge. An example of the radiological measurements is shown in Fig. 1. Radiological parameters relevant for DDH were measured by a resident with 4 years of experience, trained by a senior hip surgeon with 9 years of experience.

Pelvic tilt on the anterior–posterior pelvic x-rays was evaluated by the method developed by Schwarz et al.^18^. Therefore the distance (Distance A) between a line connecting both inferior edges of the sacro-iliac joint and a line connecting the upper edges of the obturator foramen as well as the distance (Distance B) between the line connecting the upper edges of the obturator foramen and a line connecting the lower edges of the obturator foramen was measured. The Pelvic tilt was calculated accordingly using the formula:

$$Pelvic\,Tilt=-\frac{\mathrm{ln}(\frac{B}{A}x\frac{1}{0.483})}{0.051}$$

### Classification of LSTV

**Figure 2:**
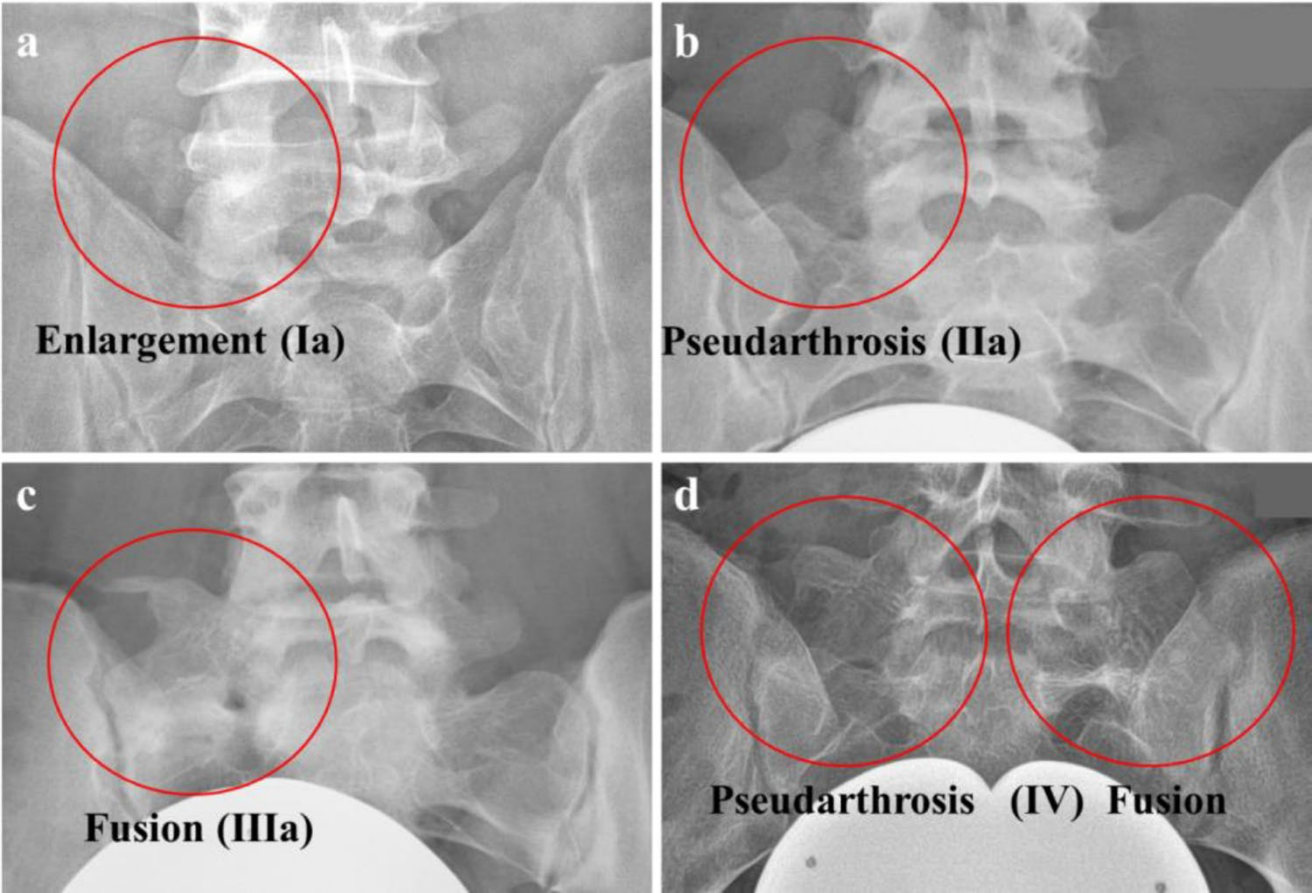
Classification of patients with LSTV according to Castellvi^12^ Dysplastic enlargement of the transversal process > 19 mm was classified as Castellvi Ia (**a**) if it appeared unilateral and as Castellvi Ib if it was detected bilateral. Patients with unilateral (IIa) (**b**) or bilateral (IIb) pseudarthrosis of the transversal process with the sacral ala were classified as Castellvi II. Patients with unilateral (IIIa) (**c**) or bilateral (IIIb) osseous fusion of the transversal process with the sacral ala were classified as Castellvi III. Patients with unilateral pseudarthrosis and contralateral osseous fusion were classified as Castellvi IV (**d**).

Preoperative radiographs were examined for LSTV by a resident with 3 years of experience guided by a senior attending orthopedic surgeon with 11 years of experience. Classification of LSTV was performed according to Castellvi as shown in Fig. 2.^12^.

### Matching

**Figure 3:**
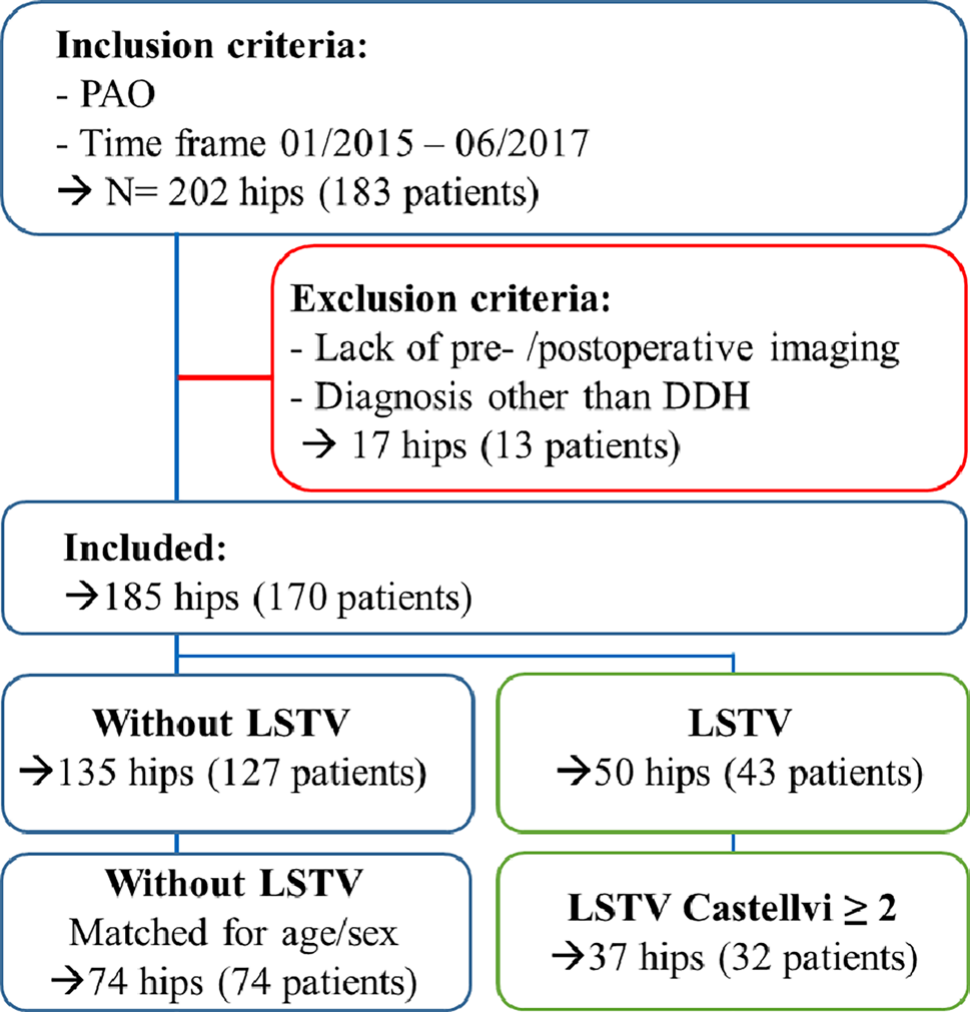
Patient selection and matching.

LSTV were detected at 50 PAO cases in 43 patients. Plain anterior–posterior radiographs as compared to Fergusson-view are known to misjudge LSTV Castellvi I frequently due to overlapping of the sacral bone with the transversal process^19^. Therefore only patients with LSTV Castellvi ≥ 2 were compared to a matched control group. The 37 hips in 32 patients who presented with a Castellvi grading ≥ 2 and thus pseudarthrosis of the transversal process or bony union of the transversal process with the sacral ala were each matched for age and sex with two patients without LSTV. A schematic overview of patient inclusion and exclusion is shown in Fig. 3.

### Assessment of PROMs

The following PROMs were assessed preoperatively and with a postoperative mean follow-up of 63.0 months (range 47–81 months): the iHOT-12 score was used to determine the health-related quality of life in younger, more active patients to evaluate treatment effects with a scale from 0 (worst) to 100 (best)^20^. The subjective hip value (SHV) was used for self-evaluation of the patient’s hip function with just one question in which 0 presents the worst and 100 the best function^21^.

### Statistics

Statistical analyses were performed using SPSSVersion 27 (IBM Corporation, New York, USA). The Kolmogorov–Smirnov test was used to test the data for normal distribution. For statistical analysis of parametric paired data, the paired T test was used. For nonparametric paired data, the Wilcoxon rank sum test was used. For testing correlations, Pearson's correlation coefficient was used for parametric data and Spearman's correlation coefficient was used for nonparametric data. The significance level was set at *p* < 0.05 for all tests.

## Results

### Demographics

The matched groups of 32 patients with LSTV and 37 PAO-procedures compared to 74 patients without LSTV (1:2 matching) did not differ significantly in age (LSTV 27.4 ± 8.3 years, control group 27.8 ± 7.7; *p* = 0.987) or sex (LSTV and control 96.9% female, *p* = 1.000). The classification of LSTV according to Castellvi is shown in Table 1.

**Table 1:** Castellvi classification of patients with LSTV.

	Castellvi I		Castellvi II		Castellvi III		Castellvi IV
	a	b	a	b	a	b	
n = 43	1	11	6	3	5	14	3
	27.9%		20.9%		44.2%		7.0%

### Effect of PAO on femoral head coverage

Lateral femoral head coverage as measured by the LCEA was significantly increased after PAO (*p* < 0.001). There was a significant effect on the Tönnis angle with a significant reduction from pre- to postoperative (*p* < 0.001). Similarly, FHEI showed a significant reduction after PAO (*p* < 0.001). The anterior coverage as measured by the AWI was increased significantly (*p* < 0.001), while the posterior coverage as measured by the PWI was significantly reduced after PAO (*p* < 0.001). Table 2 shows all pre- and postoperative femoral head coverage measurements.

**Table 2:** Femoral head coverage before and directly after PAO.

	Preoperative mean (± std.)	Postoperative mean (± std.)	*p*-value
LCEA [°]	16.4(± 6.4)	29.8(± 6.3)	** < 0.001**
TA [°]	13.2(± 7.0)	1.5(± 7.4)	** < 0.001**
FHEI [%]	23.3(± 9.5)	9.2(± 8.6)	** < 0.001**
AWI [%]	0.38 (± 0.15)	0.47 (± 0.15)	** < 0.001**
PWI [%]	0.83(± 0.19)	0.76 (± 0.23)	** < 0.001**

Values are presented as mean and standard deviation.

std., standard deviation; LCEA, lateral central edge angle; TA, Tönnis angle; FHEI, femoral head extrusion index; AWI,  anterior wall index; PWI, posterior wall index. Significance level was set at *p* < 0.05.

Significant values are marked in bold.

### Differences between patients with LSTV and controls

Pre- and postoperative measurements of femoral head coverage for patients with and without LSTV are shown in Table 3. The matched collective of 37 hips with LSTV did not differ significantly from the control group for pre- (*p* = 0.664) or postoperative (*p* = 0.660) LCEA. However, patients with LSTV had significantly increased posterior coverage compared to the control group both pre- (*p* = 0.025) and postoperatively (*p* = 0.042). No significant differences were detected in anterior femoral head coverage pre- (*p* = 0.374) or postoperatively (*p* = 0.747). No significant differences were found between patients with LSTV and control group for pelvic tilt pre- (*p* = 0.129) or postoperatively (*p* = 0.135).

**Table 3:** Comparison of correction of LCEA, TA, FHEI, AWI, PWI, PT between patients of LSTV and control group*.*

	Preoperative			Postoperative		
	LSTV mean (± std.)	Control mean (± std.)	*p*-value	LSTV mean (± std.)	Control mean (± std.)	*p*-value
LCEA [°]	17.3 (± 5.1)	16.4 (± 3.3)	0.664	29.6 (± 5.8)	30.5 (± 7.2)	0.660
TA [°]	12.4 (± 6.3)	11.3 (± 6.9)	0.667	− 0.4 (± 6.3)	1.3 (± 8.9)	0.389
FHEI [%]	20.9 (± 7.8)	23.1 (± 10.3)	0.886	5.9 (± 6.5)	9.7 (± 10.5)	0.055
AWI	0.37 (± 0.15)	0.39 (± 0.13)	0.374	0.45 (± 0.14)	0.49 (± 0.15)	0.747
PWI	0.94 (± 0.16)	0.82 (± 0.18)	**0.025**	0.85 (± 0.24)	0.76 (± 0.24)	**0.042**
PT	8.28 (± 6.50)	6.72 (± 5.15)	0.129	10.52 (± 6.68)	9.02 (± 4.89)	0.135

Values are presented as mean and standard deviation.

std., standard deviation; LCEA, lateral central edge angle; TA, Tönnis angle; FHEI, femoral head extrusion index; AWI, anterior wall index; PWI, posterior wall index; PT, Pelvic tilt. Significance level was set at *p* < 0.05.

Significant values are marked in bold.

### Influence of Castellvi grading on DDH

The expression of LSTV according to Castellvi showed no significant correlation with the lateral coverage of the femoral head. No significant correlations were found for the pre- (*p* = 0.609) or postoperative LCEA (*p* = 0.232), the pre- (*p* = 0.321) or postoperative TA (*p* = 0.081), or the pre- (*p* = 0.830) or the postoperative FHEI (*p* = 0.120). Similarly, there was no effect of the degree of expression of LSTV on the anterior femoral head coverage pre- (*p* = 0.850) or postoperatively (*p* = 0.641). However, the Castellvi degree of LSTV was significantly weak correlated with preoperative posterior coverage (*p* = 0.006, r = 0.200), whereas no significant correlation was detected between the severity of LSTV and PWI postoperatively (*p* = 0.097). Likewise no correlation between pelvic tilt in standing position and the Castellvi grading was observed pre- (*p* = 0.310) or postoperatively (*p* = 0.306).

### Influence of LSTV on patient reported outcome measurements

Pre- and postoperative PROMs were recorded in 20 patients with LSTV (62.5%) and 46 patients (62.2%) of the matched control group. Patients with LSTV and the matched control group did not differ significantly in any of the assessed scores pre- or postoperatively (Table 4).

**Table 4:** Comparison of PROMs pre- and postoperatively between patients with LSTV and control group.

	Preoperative			Postoperative		
	LSTV mean (± std.)	Control mean (± std.)	*p*-value	LSTV mean (± std.)	Control mean (± std.)	*p*-value
iHOT-12	40.7 (± 21.2)	39.7 (± 21.7)	0.859	74.3 (± 20.6)	69.6 (± 23.2)	0.266
SHV	40.3 (± 25.6)	42.6 (± 22.2)	0.953	79.1 (± 14.2)	80.3 (± 14.7)	1.000

Values are presented as mean and standard deviation.

std., standard deviation; iHOT-12, iHOT-12 Score; SHV, subjective hip value.

## Discussion

In our cohort of patients with DDH, LSTV was very common as 43 of 170 patients (25.3%) presented both. Thus, the rate of LSTV in our cohort was lower than the rate of 39–43% in patients with DDH reported by Sun et al.^13^. This may have resulted from the exclusive inclusion of patients with hip-spine syndrome in their study as well as from regional differences in the prevalence of LSTV^2,13^.

The study is the first that investigates the extent of femoral head coverage as well as both the radiological and clinical outcome after PAO. We demonstrated that patients presenting with both LSTV and DDH, compared to a matched control cohort presenting with sole DDH, had a significantly greater posterior femoral head coverage preoperatively, which persisted significantly greater also postoperatively. In our study, with respect to the lateral coverage defined by the LCEA and TA, there were no significant differences pre- or postoperatively between patients with LSTV and the matched control group. In our collective, the values of lateral coverage resulting from PAO are consistent with those reported and aimed for by surgical intervention^8^. Besides the lateral femoral head coverage, which is considered as an important parameter for the assessment of DDH in anterior–posterior pelvic radiographs, the anterior or posterior femoral head coverage as well as the acetabular version also contribute to the occurrence of hip pathologies^22^. The mean anterior coverage in our patient collective was within the range of 0.05–0.52 reported in the literature for both patients with and without concomitant LSTV^17,23^. However, the physiological value for anterior coverage is discussed controversially^17,23^. While Siebenrock et al. defined a mean of 0.28 AWI for patients with DDH, 0.41 for regular AWI, and 0.61 for overcoverage in a patient population with hip pain^17^, Anderson et al. defined 0.26 for patients with DDH, 0.35 as regular AWI, and 0.43 for patients with overcoverage in a hip pain-free population^23^. The mean anterior coverage in our cohort was 0.38 preoperatively and 0.47 postoperatively and thus around the value defined by Siebenrock et al. for regular anterior coverage, whereas according to the values of Anderson et al. anterior coverage was within the range of normal to overcoverage preoperatively and beyond the mean for overcoverage postoperatively. While no differences existed in anterior coverage between patients with LSTV and the matched control group, significant differences were seen in posterior coverage pre- and postoperatively. Patients with LSTV had significantly higher dorsal coverage compared to patients without LSTV. As for anterior coverage, the values reported in the literature for dorsal coverage differ significantly between patients with hip pain and asymptomatic patients^17,23^. While Siebenrock et al. reported a PWI of 0.81 for patients with DDH, a normal posterior coverage with a PWI of 0.91, and overcoverage of 1.15^17^, Anderson et al. reported significantly higher values in a pain-free collective of 1.03 for patients with DDH, normal posterior coverage with a PWI of 1.13, and overcoverage with an average of 1.22^23^. Our control group with hip pain had a mean PWI of 0.82, which was very close to the values defined by Siebenrock et al. for patients with DDH. Patients with LSTV had a normal to reduced dorsal coverage, depending on the used classification according to Siebenrock et al. or Anderson et al.^17,23^. However, a significantly higher dorsal coverage of patients with LSTV and DDH compared to the control group with sole DDH was detected, which was within the range of regular posterior coverage defined by Siebenrock et al.^17^.

In the anterior–posterior radiographic examination in standing position after PAO, anterior coverage was significantly increased while dorsal coverage was significantly decreased in our patient collective. On the one hand this this reduction in anteversion in anterior–posterior standing radiographs might be due to anterior tilting of the acetabulum to reduce anterior undercoverage. On the other hand, as prescribed by Schwarz et al. a rotation in coronal plane increases besides lateral femoral head coverage also the anterior femoral head coverage significantly^24^. However, postoperative imaging in standing position was performed within 7 days after surgery, requiring no weight-bearing of the operated hip. This may have resulted in reduced hip extension and concomitant increased pelvic tilting in upright standing. In this regard Dandachli et al. reported a reduction of anteversion ranging from 2.5° to 5° for every 5° of pelvic forward tilting^25^. Long-term follow-up data of PAO indicate that insufficient anterior femoral head coverage is associated with earlier osteoarthritis and necessity of conversion to hip arthroplasty^26,27^. As shown by Fujii et al. the acetabular rotation, which is defined as the acetabular tilt, strongly influences the femoral head coverage in patients with DDH. The clockwise (anterior) rotation of the acetabulum is accompanied by a decrease in posterior femoral head coverage, while the counterclockwise (posterior) acetabular rotation is associated with an increase in acetabular anteversion and decreased anterior femoral head coverage^28^. Patients with LSTV have an increased posterior coverage compared to the control group, which can be classified as normal (Siebenrock) to dysplastic (Anderson) posterior coverage^17,23^. Therefore, anterior undercoverage could be addressed by more pronounced ventral tilting of the acetabulum in patients with high acetabular anteversion for optimizing three dimensional coverage with reduced risk of posterior undercoverage. Such a configuration with a strong native acetabular anteversion, which is defined by higher discrepancies with low AWI and high PWI are more frequent found in patients with LSTV in our collective. However, acetabular retroversion should be avoided because an acetabular anteversion of < 10° as well as overcorrection of the femoral head coverage in any direction, may result in iatrogenic femoro-acetabular impingement, which is associated with PAO failure^27,29^. Despite that, differences in posture and pelvic tilt between supine and load bearing positions must be respected during acetabular reorientation during PAO, as a decreased posterior tilt in upright standing position with concomitant reduced anterosuperior acetabular coverage in the standing position was detected for patients with DDH by Tachibana et al.^30^.

No differences in pelvic tilt were detected between patients with LSTV and control group. These findings go in line with the results reported by Verhaegen et al. whom didn’t find any differences in pelvic tilt in upright standing and sitting position in a cohort of patients with LSTV compared to a control group^31^. Therefore, even if pelvic tilt is a main influencing factor of acetabular version, differences in acetabular orientation in patients with LSTV might be attributed to differences in pelvic geometry and less to differences in posture, as previously reported by Haffer et al.^2^.

At follow-up, no significant differences were found in pre- or postoperative PROMs between patients with LSTV and the control group. The outcome values of iHOT-12 after surgery were 71.0 in our population, and therefore ranged between the values reported by Fan et al. of 87.1 and 60.9 reported by Holleyman et al. The differences may result from different baseline characteristics. Whereas Holleyman et al. reported a preoperative value of 30.0 for the iHOT-12, the patient population of Fan et al. had a preoperative iHOT-12 value of 60.9, whereas our patient cohort presented 40.0. The literature reports an increase in iHOT-12 around 30 points for PAO in patients with DDH, as we found an increase of 31.0 points^32,33^. As an increase of 13 points was assessed as the minimal clinical important difference in iHOT-12 score by Nwachukwu et al. both the control group as well as the LSTV group exceeded the threshold for minimal clinical difference^34^. Therefore, for both patients with LSTV and DDH as well as patients with sole DDH the PAO procedure leads to a clinical relevant improvement of the hip.

Some limitations of our study must be noted. By examining plain AP pelvic radiographs, there is a risk of overestimating anterior coverage and underestimating posterior coverage compared with CT imaging^35^. Similarly, based on the radiographic imaging assessed, it is not possible to predict a numerical acetabular version pre- or postoperatively. Specifically, due to postoperative limited weight-bearing, increased pelvic tilting may have resulted, which could have influenced the measurement of anterior and posterior coverage and misled to the assumption of acetabular retroversion. These short postoperative changes in pelvic tilt diminish over time as recently published by Vuillemin et al. to the preoperative level, therefore the reported absolute changes in acetabular orientation by surgery might not be representative for the long-term follow-up^36^. Pelvic radiographs tend to misjudge LSTV, whereas CT imaging or radiography with Ferguson-view is the standard imaging for detection of LSTV^19^. However, performing perioperative CT imaging in otherwise young healthy patients is not reasonable, which is why our study represents daily clinical practice. PROMs were assessed at a mean follow-up of 63.0 months postoperatively and were not obtained in all patients, which may have biased the evaluation of postoperative outcome. Our study does not reflect long-term results in which conversion to hip arthroplasty would be considered as the main adverse long-term outcome of PAO. For the fixation method of the acetabular fragment K-wire fixation or screw fixation were chosen according to the preferences of the surgeon, however the literature presents no differences in fixation stability between the two methods^37^.

## Conclusion

Patients with both DDH and LSTV have a significantly higher dorsal femoral head coverage compared to patients with sole DDH. To avoid anterior undercoverage, which is a significant predictor for premature conversion to hip arthroplasty after PAO, a more pronounced ventral tilting might be performed in those patients with both DDH and LSTV with extensive acetabular anteversion, which might be accompanied by prominent posterior wall sign. However, care must be taken not to create iatrogenic pincer impingement due to acetabular retroversion. Patients with LSTV reported similar functional outcomes and activity after PAO as the control group. Therefore, even for patients with concomitant LSTV, which are frequent with one-fourth in our cohort, PAO is an efficient treatment option to improve clinical symptoms caused by DDH.

## Data Availability

The authors confirm that the data supporting the findings of this study are available within the article.
